# Regulation of capillary hemodynamics by K_ATP_ channels in resting skeletal muscle

**DOI:** 10.14814/phy2.14803

**Published:** 2021-05-01

**Authors:** Daniel M. Hirai, Ayaka Tabuchi, Jesse C. Craig, Trenton D. Colburn, Timothy I. Musch, David C. Poole

**Affiliations:** ^1^ Department of Health and Kinesiology Purdue University West Lafayette Indiana USA; ^2^ Department of Kinesiology Kansas State University Manhattan Kansas USA; ^3^ Department of Engineering Science University of Electro‐Communications Tokyo Japan; ^4^ Department of Internal Medicine University of Utah Salt Lake City Utah USA; ^5^ Geriatric Research Education and Clinical Center Veterans Affairs Medical Center Salt Lake City Utah USA; ^6^ Department of Anatomy and Physiology Kansas State University Manhattan Kansas USA

**Keywords:** ATP‐sensitive K^+^ channel, blood flow, intravital microscopy, microcirculation, red blood cell

## Abstract

ATP‐sensitive K^+^ channels (K_ATP_) have been implicated in the regulation of resting vascular smooth muscle membrane potential and tone. However, whether K_ATP_ channels modulate skeletal muscle microvascular hemodynamics at the capillary level (the primary site for blood‐myocyte O_2_ exchange) remains unknown. We tested the hypothesis that K_ATP_ channel inhibition would reduce the proportion of capillaries supporting continuous red blood cell (RBC) flow and impair RBC hemodynamics and distribution in perfused capillaries within resting skeletal muscle. RBC flux (*f*_RBC_), velocity (*V*_RBC_), and capillary tube hematocrit (Hct_cap_) were assessed via intravital microscopy of the rat spinotrapezius muscle (*n* = 6) under control (CON) and glibenclamide (GLI; K_ATP_ channel antagonist; 10 µM) superfusion conditions. There were no differences in mean arterial pressure (CON:120 ± 5, GLI:124 ± 5 mmHg; *p* > 0.05) or heart rate (CON:322 ± 32, GLI:337 ± 33 beats/min; *p* > 0.05) between conditions. The %RBC‐flowing capillaries were not altered between conditions (CON:87 ± 2, GLI:85 ± 1%; *p* > 0.05). In RBC‐perfused capillaries, GLI reduced *f*_RBC_ (CON:20.1 ± 1.8, GLI:14.6 ± 1.3 cells/s; *p* < 0.05) and *V*_RBC_ (CON:240 ± 17, GLI:182 ± 17 µm/s; *p* < 0.05) but not Hct_cap_ (CON:0.26 ± 0.01, GLI:0.26 ± 0.01; *p* > 0.05). The absence of GLI effects on the %RBC‐flowing capillaries and Hct_cap_ indicates preserved muscle O_2_ diffusing capacity (DO_2_m). In contrast, GLI lowered both *f*_RBC_ and *V*_RBC_ thus impairing perfusive microvascular O_2_ transport (Q̇m) and lengthening RBC capillary transit times, respectively. Given the interdependence between diffusive and perfusive O_2_ conductances (i.e., %O_2_ extraction∝DO_2_m/Q̇m), such GLI alterations are expected to elevate muscle %O_2_ extraction to sustain a given metabolic rate. These results support that K_ATP_ channels regulate capillary hemodynamics and, therefore, microvascular gas exchange in resting skeletal muscle.

## Graphical abstract

Superfusion of the rat spinotrapezius muscle with the sulfonylurea glibenclamide (GLI) was used to locally inhibit K_ATP_ channels *in vivo*. GLI reduced both red blood cell (RBC) flux and velocity thereby impairing perfusive microvascular O_2_ transport and lengthening RBC capillary transit times, respectively. These results support that K_ATP_ channels regulate capillary hemodynamics and microvascular gas exchange in resting skeletal muscle.

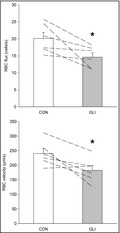

## 1 INTRODUCTION

The skeletal muscle capillary vascular bed provides the largest surface area for gas and substrate exchange within the body (Hirai et al., 2019; Poole et al., 2011; Poole, 2019). Regulation of skeletal muscle capillary hemodynamics is mediated primarily at the arteriolar level (Joyner & Casey, 2015; Kindig & Poole, 2001; Laughlin et al., 2012; Segal, 2005). Several complex and often interacting mechanisms are responsible for alterations in arteriolar resistance and thus vascular conductance. Among these processes, K^+^ channels constitute the dominant ion conductance of the vascular smooth muscle cell determining membrane potential, contractile activity, and thus vascular tone (Foster & Coetzee, 2016; Jackson, 2017; Tykocki et al., 2017).

ATP‐sensitive K^+^ (K_ATP_) channels constitute one of the, at least, five distinct classes of K^+^ channels regulating vascular smooth muscle function (Foster & Coetzee, 2016; Jackson, 2017; Tykocki et al., 2017). Inhibition (closing) of K_ATP_ channels decreases K^+^ efflux leading to depolarization of the smooth muscle cell. Voltage‐gated Ca^2+^ channels then transduce membrane depolarization into increased Ca^2+^ influx promoting vascular muscle contraction (i.e., vasoconstriction). *In vitro* and *in vivo* studies have thus used pharmacological K_ATP_ channel inhibition to evaluate its potential role in setting vascular tone and, consequently, tissue perfusion. Sulfonylureas such as glibenclamide (GLI), which are employed clinically in the treatment of non‐insulin‐dependent diabetes (Montvida et al., 2018), represent the most frequently used class of K_ATP_ channel inhibitors. To date, however, there is a lack of agreement regarding the role of K_ATP_ channels in the regulation of resting vascular tone and blood flow within skeletal muscle (Foster & Coetzee, 2016; Jackson, 2017; Tykocki et al., 2017). K_ATP_ channel inhibition with GLI has produced conflicting effects on resting skeletal muscle arteriolar diameter (Hammer et al., 2001; Hodnett et al., 2008; Jackson, 1993; Lu et al., 2013; Murrant & Sarelius, 2002; Saito et al., 1996; Xiang & Hester, 2009) and bulk blood flow (Bank et al., 2000; Colburn, Holdsworth, et al., 2020; Duncker et al., 2001; Farouque & Meredith, 2003a, 2003b,2003a, 2003b; Holdsworth et al., 2015; Vanelli & Hussain, 1994). Importantly, the functional role of K_ATP_ channels within the skeletal muscle capillary network (i.e., the primary site for blood‐myocyte O_2_ exchange) remains to be determined. Resolution of the mechanisms modulating capillary red blood cell (RBC) hemodynamics has direct relevance to transcapillary O_2_ flux and cellular energetic status.

The purpose of this study was to evaluate the regulation of capillary RBC hemodynamics by K_ATP_ channels in resting skeletal muscle. Using intravital microscopy techniques combined with the rat spinotrapezius preparation, we tested the hypothesis that acute local K_ATP_ channel inhibition with GLI would impair key determinants of capillary diffusive and perfusive O_2_ conductances. Specifically, GLI was anticipated to reduce the proportion of capillaries supporting continuous RBC flow and impair microvascular hemodynamics and distribution (i.e., RBC flux, velocity, and capillary tube hematocrit) in perfused capillaries within resting skeletal muscle. Confirmation of these hypotheses would support a role for K_ATP_ channels in the regulation of skeletal muscle capillary hemodynamics (and thus microvascular gas exchange) under resting conditions.

## 2 METHODS

Intravital microscopy was used to evaluate the *in vivo* spinotrapezius muscle microcirculation in healthy young male Sprague–Dawley rats (*n* = 6; ~3–4 months old; 386 ± 26 g). Rats were maintained in accredited facilities (Association for the Assessment and Accreditation of Laboratory and Animal Care) under a 12:12 h light–dark cycle with food and water provided *ad libitum*. All experimental procedures and protocols were approved by the Institutional Animal Care and Use Committee of Kansas State University and followed the guidelines established by the National Institutes of Health.

### 2.1 Surgical procedures

#### 2.1.1 Anesthesia and catheter placement procedures

All rats were anesthetized initially with a 5% isoflurane‐O_2_ mixture and maintained subsequently on 2–3% isoflurane‐O_2_ (Butler Animal Health Supply). Anesthetized rats were kept on a heating pad to maintain core temperature at ~37–38°C as measured via a rectal probe. The right carotid artery was cannulated (PE‐10 connected to PE‐50; BD IntraMedic Polyethylene Tubing) for continuous measurements of mean arterial pressure and heart rate (MAP and HR, respectively; PowerLab; ADInstruments). The caudal artery was cannulated (PE‐10 connected to PE‐50) for infusion of anesthetic agents. Isoflurane inhalation was then discontinued progressively and rats were kept under anesthesia with pentobarbital sodium (50 mg/kg i.a.) throughout the remainder of the experiment. Anesthesia level was monitored at frequent and regular intervals via the toe‐pinch and blink reflexes and supplemented as necessary (0.02–0.05 ml of 50 mg/ml pentobarbital sodium diluted in ~0.2 ml of heparinized saline).

#### 2.1.2 Intravital microscopy preparation

The rat spinotrapezius muscle was used for the microcirculatory studies herein because a) its exteriorization allows for clear visualization of capillary structure and hemodynamics via light transmission microscopy (Gray, 1973; Poole et al., 1997); b) it can be exteriorized without neural or substantial vascular disruption (Bailey et al., 2000; Gray, 1973; Poole et al., 1997); c) a physiological sarcomere length can be maintained throughout the experimental protocol (thereby preventing muscle overstretching and adverse microcirculatory effects) (Poole et al., 1997; Welsh & Segal, 1996); and d) its mixed fiber‐type composition and oxidative capacity are similar to those of the human quadriceps (Delp & Duan, 1996; Leek et al., 2001), thus representing a useful analog of human locomotor muscle. Following catheter placement procedures, the left spinotrapezius was exposed and exteriorized as described previously (Bailey et al., 2000; Gray, 1973; Poole et al., 1997) with minimal fascial removal to limit tissue damage and microcirculatory disturbances (Mazzoni et al., 1990). Briefly, the caudal end of the muscle was isolated from its origin and sutured at equidistant points to a horseshoe‐shaped manifold. The rat was then placed on a water circulation‐heated (38°C) Lucite platform and the manifold secured with the ventral aspect of the muscle reflected upwards for microscopic observation. The preparation was frequently superfused with Krebs–Henseleit bicarbonate‐buffered solution (2.0 mM CaCl_2_, 2.4 mM MgSO_4_, 4.7 mM KCl, 22 mM NaHCO_3_, 131 mM NaCl; pH 7.4; equilibrated with 5% CO_2_ and 95% N_2_ at ~38°C) and exposed surrounding tissue covered with Saran wrap (Dow Brands). As noted earlier, the spinotrapezius was maintained at physiological sarcomere length (2.6 ± 0.1 µm) to prevent stretch‐induced reductions in capillary blood flow (Poole et al., 1997; Welsh & Segal, 1996).

### 2.2 Experimental protocol

#### 2.2.1 Intravital video microscopy

Microcirculatory fields located midway between arteriolar and venular ends within the mid‐caudal region of the spinotrapezius that provided optimal clarity were selected randomly for the study. Microcirculatory images were obtained via a bright‐field microscope (Nikon Eclipse E600‐FN) equipped with a non‐contact illuminated lens (x40, numerical aperture 0.8) and viewed in real time on a high‐resolution color monitor (Sony Trinitron PVM‐1954Q) under a final magnification of x1,184 (as confirmed by initial calibration of the system with a stage micrometer; MA285; Meiji Techno). Images were recorded by a video camera (JVC KY‐F55B) and stored on a computer for subsequent offline analyses.

#### 2.2.2 Experimental design

Once the spinotrapezius muscle was positioned on the platform, a quiescent period of at least 15 min was allowed before any data were acquired. Intravital microscopy recordings were then made under two separate superfusion conditions: control (Krebs–Henseleit; CON) and K_ATP_ channel inhibition (glibenclamide; GLI). GLI was the last treatment due to its long‐lasting effects (Thomas et al., 1997) and the possibility of incomplete washout with Krebs–Henseleit. All superfusion solutions were maintained at approximately 38°C. The spinotrapezius was superfused with each solution for 3 min (average flow rate of 1 ml/min) followed by a 30 min equilibration period as employed by previous microcirculatory studies (Hodnett et al., 2008; Jackson, 1993; Lu et al., 2013; Murrant & Sarelius, 2002; Saito et al., 1996). Microcirculatory fields were chosen randomly from each rat (based on clear visualization of sarcomeres, fibers, and capillaries) and each recorded for ~1–1.5 min. At the end of the experimental protocol, rats were killed with intra‐arterial pentobarbital sodium overdose (100 mg/kg) followed by pneumothorax.

#### 2.2.3 Acute local K_ATP_ channel inhibition

The pharmacological sulphonylurea derivative GLI (494 g/mol; 5‐chloro‐*N*‐[4‐(cyclohexylureidosulfonyl)phenethyl]‐2‐methoxybenzamide; Sigma‐Aldrich; St. Louis, MO) was used to inhibit K_ATP_ channels via superfusion (topical application) of the spinotrapezius muscle. GLI stock solutions were made fresh daily with the solvent dimethyl sulfoxide (DMSO) and diluted with control (Krebs–Henseleit) superfusate. The final working superfusate had a GLI concentration of 10 µM and contained <0.01% DMSO. This GLI concentration was based on previous microcirculatory research using similar drug delivery methods with the rat spinotrapezius and hamster cheek pouch and cremaster muscles (Cohen & Sarelius, 2002; Hammer et al., 2001; Hodnett et al., 2008; Jackson, 1993; Lu et al., 2013; Murrant & Sarelius, 2002; Saito et al., 1996; Xiang & Hester, 2009). Previous studies have shown that ≤0.05% DMSO solutions have no significant effects on resting skeletal muscle arteriolar diameter or reactivity (Cohen et al., 2000; Jackson, 1993) or K_ATP_ channel currents in the absence or presence of ATP (Mele et al., 2014; Tricarico et al., 2006). Recent reports from our laboratory have shown no differences in arterial blood PO_2_, PCO_2_, O_2_ saturation, hematocrit, pH, [lactate] or [glucose] following superfusion of the rat spinotrapezius muscle with K_ATP_ channel agonists (pinacidil; 5 mg/kg) and antagonists (GLI; 5 mg/kg) (Holdsworth et al., 2017).

### 2.3 Analysis of muscle capillary hemodynamics

Within each preparation, only those microcirculatory fields with the best overall clarity were selected for further examination via frame‐by‐frame techniques (30 frames/s; Dartfish) as described previously (Kindig et al., 1999, 2002; Poole et al., 1997). Sarcomere length was determined from sets of 10 consecutive in‐register sarcomeres (i.e., distance between 11 consecutive A‐bands) measured in parallel to the muscle fiber longitudinal axis. This procedure was repeated 3 times where sarcomeres were visible to obtain a mean sarcomere length for each field. Comparisons between control and GLI conditions were made within the same microcirculatory field for each rat and, whenever possible, between the same capillaries (which occurred in ~47% of cases; i.e., 14 of 30 vessels). The percentage of RBC‐perfused vessels was established as (no. of capillaries supporting RBC flow/total no. of visible capillaries per field) × 100. Vessels demonstrating impeded or stopped flow (i.e., stationary or no visible RBC flow) for ≥10 s were regarded as non‐flowing capillaries. Five capillaries supporting continuous RBC flow were selected randomly for hemodynamics analysis from each microcirculatory field based on clarity. For all capillaries in which hemodynamics was evaluated and where the capillary endothelium was clearly visible on both sides of the lumen, capillary luminal diameter (*d*_cap_) was measured at two random sites per capillary (2–3 measurements/site). RBC velocity (*V*_RBC_) was determined by following the RBC path length over several frames and for the maximum capillary length over which the cells remained in crisp focus. RBC flux (*f*_RBC_) was measured by counting the number of cells in a capillary passing an arbitrary point over several frames. These measurements were performed three times per capillary. For each capillary in which hemodynamics data were measured, capillary tube hematocrit (Hct_cap_) was calculated as follows:

(1) $${\text{Hct}}_{\text{cap}}=\left({\text{vol}}_{\text{RBC}}\times f_{\text{RBC}}\right)/\left[\pi \times {\left(d_{\text{cap}}/2\right)}^{2}\times V_{\text{RBC}}\right]$$

where vol_RBC_ is RBC volume and assumed to be 61 µm^3^ (Altman & Dittmer, 1974) and capillaries were approximated as circular in cross section (Desjardins & Duling, 1990; Kindig et al., 1998).

### 2.4 Statistical analyses

Statistical analyses were performed using a commercially available software package (SigmaPlot 11.2; Systat Software). Data distribution was assessed via the Shapiro–Wilk test for normality. Central hemodynamics (MAP and HR) and spinotrapezius muscle capillary structure (*d*_cap_) and hemodynamics (%RBC‐flowing capillaries, *f*_RBC_, *V*_RBC_, and Hct_cap_) data were compared between control and GLI conditions using paired Student's *t*‐tests. Coefficients of variation were calculated as (SD/mean) × 100 and compared between control and GLI conditions using the methods described by Forkman (2009). Pearson's product‐moment correlations and linear regression analyses were used to examine relationships between *f*_RBC_ and *V*_RBC_. Linear regression slope comparisons were performed as described by Zar (1984). Significance was accepted at *p* < 0.05. Data are reported as mean ± SE.

## 3 RESULTS

### 3.1 Central hemodynamics

As expected based on the topical drug delivery method employed herein (i.e., local superfusion of the spinotrapezius muscle), no differences in MAP (CON: 120 ± 5, GLI: 124 ± 5 mmHg; *p* > 0.05) or HR (CON: 322 ± 32, GLI: 337 ± 33 beats/min; *p* > 0.05) were observed between conditions.

### 3.2 Spinotrapezius muscle capillary structure and hemodynamics

**FIGURE 1:**
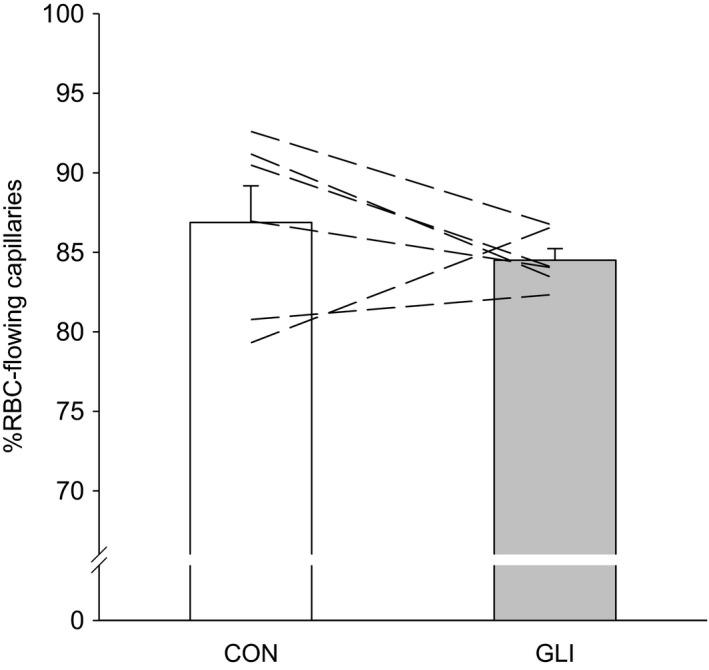
Percentage of capillaries supporting red blood cell (RBC) flow during control (CON; *n* = 6) and K_ATP_ channel inhibition (glibenclamide, GLI; *n* = 6) conditions. Dashed lines represent individual muscle data

**FIGURE 2:**
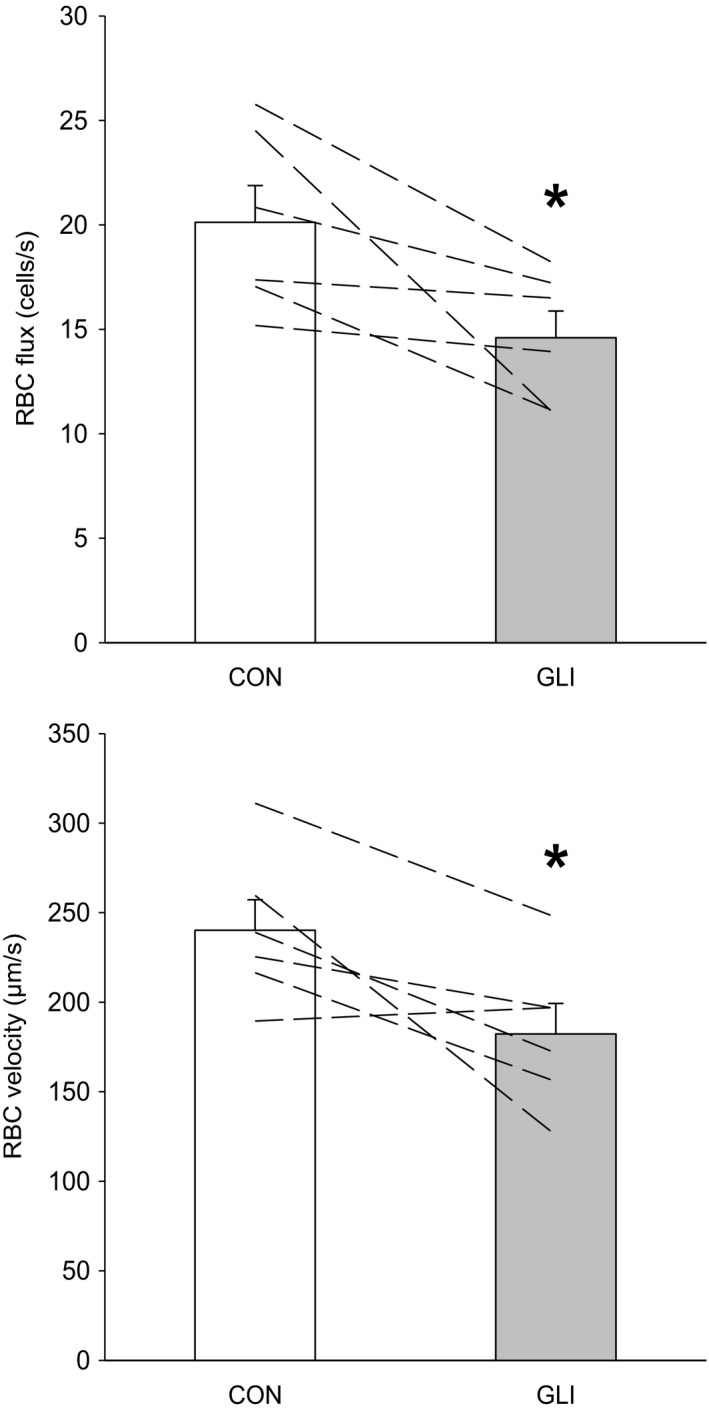
Capillary red blood cell flux (*top panel*) and velocity (*bottom panel*) during control (CON; *n* = 6) and K_ATP_ channel inhibition (glibenclamide, GLI; *n* = 6) conditions. Dashed lines represent individual muscle data. *p < 0.05 vs. CON

**FIGURE 3:**
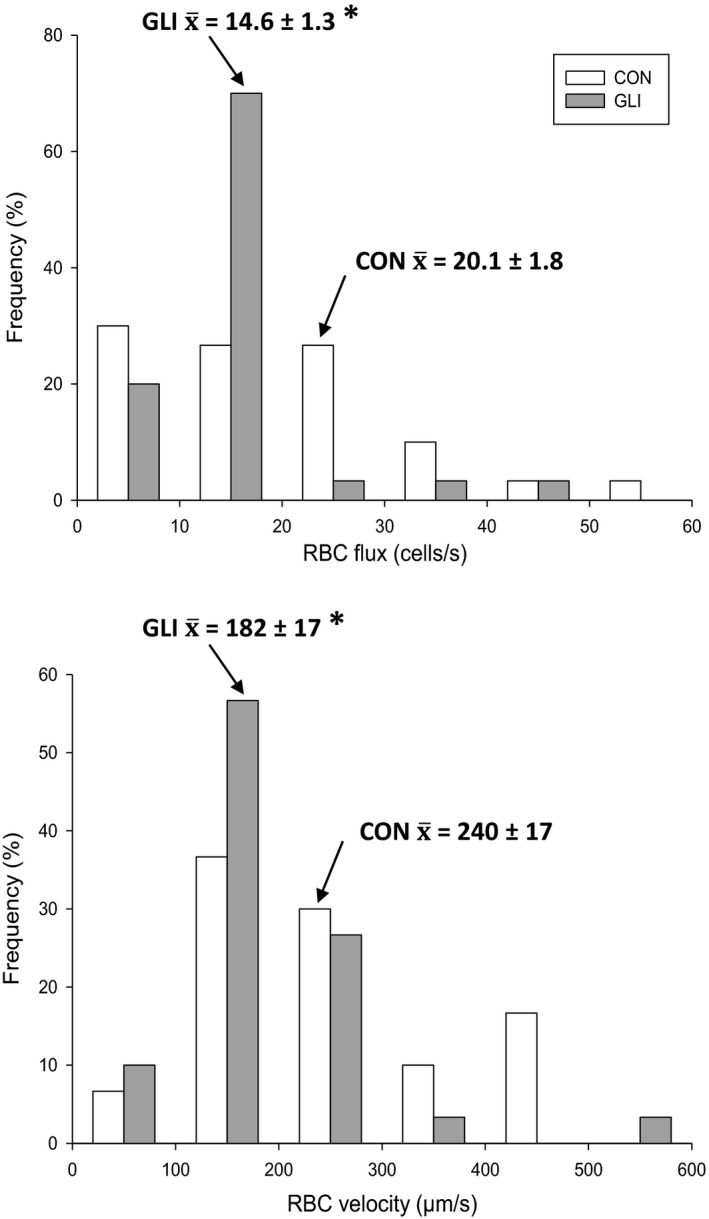
Relative frequency histograms of capillary red blood cell flux (*top panel*) and velocity (*bottom panel*) during control (CON; *n* = 6) and K_ATP_ channel inhibition (glibenclamide, GLI; *n* = 6) conditions. Arrows show mean values. *p < 0.05 vs. CON

The percentage of capillaries supporting continuous RBC flow was not altered between conditions (Figure 1; *p* > 0.05). Capillaries subjected to hemodynamics analysis supported RBC flow prior to, and continued to do so, following K_ATP_ inhibition (i.e., during CON and GLI, respectively). In these RBC‐flowing capillaries, GLI reduced both *f*_RBC_ and *V*_RBC_ (Figures 2 and 3; *p* < 0.05 for both) but not Hct_cap_ (CON: 0.26 ± 0.01, GLI: 0.26 ± 0.01; *p* > 0.05). GLI‐induced alterations in muscle capillary hemodynamics occurred in the absence of changes in *d*_cap_ between conditions (CON: 4.9 ± 0.1, GLI: 4.9 ± 0.1 µm; *p* > 0.05). Frequency histograms of capillary *f*_RBC_ and *V*_RBC_ are shown in Figure 3. Microvascular blood flow heterogeneity, as evaluated by the individual capillary coefficient of variation, was not different between conditions with respect to *f*_RBC_ (CON: 61.8, GLI: 57.4%; *p* > 0.05), *V*_RBC_ (CON: 49.4, GLI: 47.8%; *p* > 0.05) or Hct_cap_ (CON: 23.6, GLI: 22.3%; *p* > 0.05). Similar results were found when comparing heterogeneity between microvascular fields in terms of *f*_RBC_ (CON: 21.4, GLI: 21.6%; *p* > 0.05), *V*_RBC_ (CON: 17.4, GLI: 22.9%; *p* > 0.05), and Hct_cap_ (CON: 13.5, GLI: 11.1%; *p* > 0.05).

**FIGURE 4:**
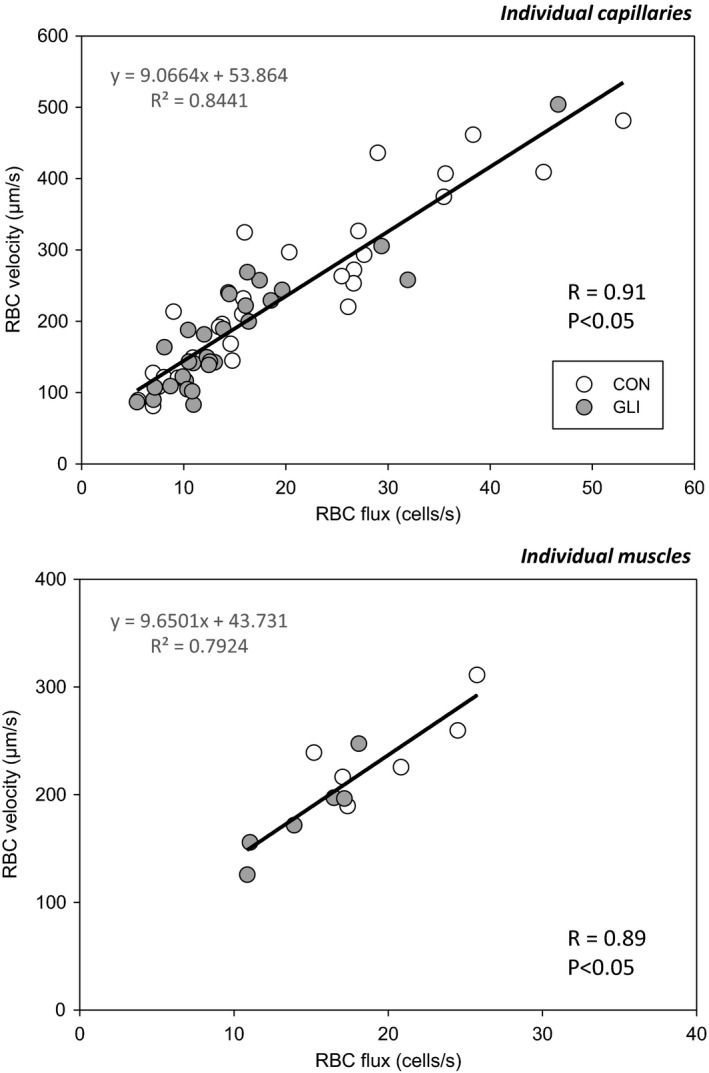
Individual and mean capillary relationships (*top* and *bottom panels*, respectively) between red blood cell flux and velocity during control (CON) and K_ATP_ channel inhibition (glibenclamide, GLI) conditions. Each data point represents a single capillary in the *top panel* (*n* = 30) and the average value for all capillaries within a single muscle (*n* = 6) in the *bottom panel*

Consistent with the lack of changes in Hct_cap_ with GLI (and the relationship described in Eq. 1 above), no significant differences were observed in the slope of the *f*_RBC_/*V*_RBC_ relationship between conditions (individual capillaries; CON: 8.75, GLI: 9.44; individual muscles; CON: 7.46, GLI: 12.25; *p* > 0.05 for both). Therefore, Figure 4 presents linear correlations and regression analyses for the combined CON and GLI data. Capillary *f*_RBC_ and *V*_RBC_ were significantly correlated in both individual capillaries and individual muscles (Figure 4, *top* and *bottom panels*, respectively) thus illustrating their proportional reduction with GLI.

## 4 DISCUSSION

This investigation examined, for the first time, the regulation of capillary hemodynamics by K_ATP_ channels in resting skeletal muscle. Superfusion of the rat spinotrapezius with the sulfonylurea GLI was used to locally inhibit K_ATP_ channels *in vivo*. The principal novel findings are as follows: a) inconsistent with our hypothesis, GLI did not reduce the proportion of capillaries supporting continuous RBC flow or Hct_cap_; and b) consistent with our hypothesis, GLI lowered microvascular blood flow (i.e., both *f*_RBC_ and *V*_RBC_) in perfused capillaries. These data suggest that K_ATP_ channels, via modulation of arteriolar vascular conductance, regulate microvascular hemodynamics at the capillary level (the primary site for blood‐myocyte O_2_ diffusion) (Hirai et al., 2019; Poole et al., 2011; Poole, 2019) in resting skeletal muscle.

### 4.1 Intravital microscopy preparation

Surgical exteriorization of the rat spinotrapezius muscle was performed as described previously (Bailey et al., 2000; Gray, 1973; Poole et al., 1997) with minimal fascial disturbance to curtail tissue damage and related microcirculatory consequences. Accordingly, previous studies from our laboratory indicate that the surgical exteriorization requisite for transmission intravital microscopy as performed herein does not impair the microvascular integrity or responsiveness of the spinotrapezius muscle (Bailey et al., 2000).

In the present investigation, spinotrapezius sarcomere length was set at physiological values (2.6 ± 0.1 µm) to prevent stretch‐induced capillary luminal diameter reductions (Poole et al., 1997) and/or sympathetically mediated decreases in arteriolar blood flow (Welsh & Segal, 1996; cf. Kindig & Poole, 2001). Capillary structure (*d*_cap_) and hemodynamics (%RBC‐flowing vessels, *f*_RBC_, *V*_RBC_, and Hct_cap_) data obtained herein during the control condition are consistent with those published previously in the resting rat spinotrapezius and hamster cheek pouch and cremaster muscles (Copp et al., 2009; Kano et al., 2005; Kindig et al., 1998, 1999, 2002; Kindig & Poole, 2001; Poole et al., 1997; Richardson et al., 2003; Russell et al., 2003; Sarelius & Duling, 1982). Our data are also consistent with the well‐documented lower than systemic Hct_cap_ values found in resting skeletal muscle (Poole et al., 2011; Poole, 2019). Based on both theoretical and empirical studies, potential mechanisms for the systemic versus capillary tube hematocrit difference include a) the presence of the endothelial surface layer (i.e., the often termed “glycocalyx”); b) the Fahraeus effect; and c) plasma skimming at arteriolar bifurcations (Desjardins & Duling, 1990; Ellsworth et al., 2009; Pries et al., 1986; Secomb et al., 1998).

As reviewed recently (Poole et al., 2011; Poole, 2019), a compelling body of evidence demonstrates that the vast majority of skeletal muscle capillaries support continuous RBC perfusion at rest. Our data are consistent with this notion showing that ~85% of capillaries supported continuous *f*_RBC_ during both CON and GLI conditions (Figure 1).

### 4.2 Local K_ATP_ channel inhibition with GLI superfusion

As expected, GLI‐induced alterations in microvascular hemodynamics (*f*_RBC_ and *V*_RBC_; Figure 2) occurred in the absence of changes in *d*_cap_ between conditions. This suggests increased vascular resistance at sites upstream of the capillary bed consistent with the well‐established arteriolar control of skeletal muscle blood flow (Joyner & Casey, 2015; Kindig & Poole, 2001; Laughlin et al., 2012; Segal, 2005). Our data are thus in agreement with some, but not all, reports of decreased arteriolar diameter (Hammer et al., 2001; Hodnett et al., 2008; Jackson, 1993; Lu et al., 2013; Murrant & Sarelius, 2002; Saito et al., 1996; Xiang & Hester, 2009) and bulk blood flow (Bank et al., 2000; Colburn, Weber, et al., 2020; Duncker et al., 2001; Farouque & Meredith, 2003a, 2003b,2003a, 2003b; Holdsworth et al., 2015; Vanelli & Hussain, 1994) following K_ATP_ channel inhibition in the resting skeletal muscle. Potential reasons for this discrepancy include species differences, experimental models, GLI doses, K_ATP_ channel density distribution, compensatory vasodilation by redundant pathways, muscle fiber type, and arteriolar size and branch order. Although providing an invaluable framework for understanding the control of vascular tone and tissue perfusion, previous measurements of arteriolar diameter and bulk blood flow responses with GLI presented no information concerning the distribution of microvascular flow within the capillary network. Moreover, it was not known whether K_ATP_ channel inhibition would impact uniformly RBC‐perfused capillaries or whether the proportion of RBC‐perfused capillaries could be reduced. Evaluation of capillary hemodynamics within those vessels supporting continuous RBC flow is critical to identifying how K_ATP_ channels may modulate blood‐myocyte O_2_ and substrate exchange. This is especially true when considering that the capillary network provides the largest surface area for gas and substrate exchange within the skeletal muscle microcirculation (Hirai et al., 2019; Poole et al., 2011; Poole, 2019).

The potential for K_ATP_ channels to modulate skeletal muscle O_2_ exchange must be considered in light of the interdependence between diffusive and perfusive conductances setting fractional O_2_ extraction (Roca et al., 1992):

(2) $$\%O_{2}\mathrm{extraction}=1‐e^{‐{\mathrm{DO}}_{2}m/\beta \dot{Q}m}\;$$

where DO_2_m is muscle O_2_ diffusing capacity, β is the slope of the O_2_ dissociation curve in the physiologically relevant range, and Q̇m is muscle blood flow. Theoretical models of skeletal muscle O_2_ exchange indicate that DO_2_m is determined by structural (capillary‐to‐fiber ratio, capillary length, and *d*_cap_) and functional (Hct_cap_ at constant arterial O_2_ saturation) elements (Federspiel & Popel, 1986; Groebe & Thews, 1990). Capillary‐to‐fiber ratio, capillary length, and β are not expected to be affected by acute GLI treatment as employed herein. Moreover, we observed no differences in *d*_cap_ or Hct_cap_ between CON and GLI. It thus seems that K_ATP_ channels do not modulate significantly DO_2_m in resting skeletal muscle. On the other hand, the current GLI‐induced reductions in *f*_RBC_ (which largely dictates Q̇m within the microcirculation) (Berg & Sarelius, 1996) are anticipated to elevate the DO_2_m/Q̇m ratio within RBC‐perfused capillaries and, therefore, necessitate higher muscle fractional O_2_ extraction for a given metabolic rate according to Eq. 2 above. Importantly, the functional consequence of lowered *f*_RBC_ with GLI (Figure 2, *top panel*) is impaired O_2_ delivery per capillary (Q̇O_2cap_). Disregarding the small amount of O_2_ dissolved in plasma (and, therefore, its relevance to capillary gas exchange), Q̇O_2cap_ can be estimated as the product of *f*_RBC_, the hemoglobin content per RBC (17 × 10^−12 ^g per RBC) (Altman & Dittmer, 1974) and the O_2_ carrying capacity of hemoglobin (at 89% saturation, assuming arterial PO_2_ = 80 mmHg in anesthetized rats) as described previously (Kindig et al., 1998):

(3) $${\dot{Q}O}_{2\mathrm{cap}}=f_{\mathrm{RBC}}\left(g\;\mathrm{Hb}\times {\mathrm{RBC}}^{‐1}\right)\left(1.34\;\mathrm{ml}\;O_{2}\times g\;{\mathrm{Hb}}^{‐1}\times 0.89\right)$$

Using the equation above, K_ATP_ inhibition with GLI superfusion of the resting spinotrapezius herein reduced mean Q̇O_2cap_ by approximately 27% from 4.08 to 2.96 × 10^−10 ^ml O_2_/s. Interestingly, this estimation is strikingly similar to the ~28% reduction in total hindlimb skeletal muscle blood flow of conscious resting rats following GLI administration (Colburn, Holdsworth, et al., 2020).

GLI‐induced alterations in *V*_RBC_ (Figure 2, *bottom panel*) underscore the potential of K_ATP_ channels to further modulate capillary gas exchange via changes in RBC transit time. Given the ~25% decrease in *V*_RBC_ with GLI observed herein and the mean capillary length within the rat spinotrapezius (i.e., 430 µm based on previous morphometric analyses by Gray, 1984), total capillary RBC residence time is calculated to increase from ~1.8 s during control to ~2.4 s following K_ATP_ channel inhibition. While it is acknowledged that these calculations may underestimate true residence time due to the actual RBC path length being somewhat longer than the anatomical path length (Sarelius, 1986), the amount of error introduced should not preferentially bias either superfusion condition. Longer RBC transit times provide the mechanistic basis for the elevated fractional O_2_ extraction expected with GLI as discussed above.

The proportional reductions in *f*_RBC_ and *V*_RBC_ with GLI were such that Hct_cap_ remained unchanged between conditions (*vide supra* its mathematical description; Eq. 1). It is interesting that the relationship between *f*_RBC_ and *V*_RBC_ in the resting skeletal muscle may also be preserved with aging and some disease states. Accordingly, previous studies from our laboratory have shown that the microvascular dysregulation characteristic of aging (Copp et al., 2009; Russell et al., 2003), type I diabetes (Kindig et al., 1998), and chronic heart failure (Kindig et al., 1999; Richardson et al., 2003) does not appear to affect resting Hct_cap_. As noted above, Hct_cap_ is a primary determinant of DO_2_m and, therefore, diffusive O_2_ conductance within the microcirculation (Federspiel & Popel, 1986; Groebe & Thews, 1990). This derives from the low O_2_ diffusivity in plasma and the particulate nature of blood, which render only the capillary surface area in close proximity to the RBC functional for diffusion at any given time. The current Hct_cap_ data thus indicate preserved capillary surface area available for diffusive O_2_ exchange within continuously RBC‐perfused vessels following K_ATP_ channel inhibition.

An important question for future studies is whether the impairments in resting capillary hemodynamics seen here after K_ATP_ inhibition are also present during muscle contractions and, if so, their potential implications for exercise tolerance. In the resting spinotrapezius, GLI lowered *f*_RBC_ and *V*_RBC_ (i.e., reduced Q̇m; Figure 2) whilst not affecting Hct_cap_ or the %RBC‐flowing capillaries (i.e., unaltered DO_2_m as noted above), thereby predisposing to higher fractional O_2_ extraction (i.e., higher DO_2_m/Q̇m ratio; Eq. 2). While there has been no prior evaluation of K_ATP_ channel inhibition on contracting muscle capillary blood flow *per se*, studies on arteriolar diameter and bulk blood flow have produced contrasting evidence regarding the role of K_ATP_ channels in functional hyperemia (Tykocki et al., 2017). Nonetheless, recent reports from our laboratory indicate that GLI lowers microvascular O_2_ partial pressures (PO_2_) as well as estimated DO_2_m in the contracting spinotrapezius and mixed gastrocnemius muscles (Colburn, Weber, et al., 2020; Holdsworth et al., 2016). Under these circumstances, intramyocyte PO_2_ is expected to fall to generate the driving pressure (ΔPO_2_) and possibly elevate intramyocyte O_2_ diffusing capacity by and by further deoxygenating myoglobin as necessary to maintain a given metabolic rate as described by Fick's law of diffusion:

(4) $${\dot{V}O}_{2}={\mathrm{DO}}_{2}m\times {\Delta \mathrm{PO}}_{2}\;$$

where V̇O_2_ corresponds to the rate of O_2_ flux, DO_2_m is the diffusing capacity as defined above and ΔPO_2_ the O_2_ partial pressure difference between the microvascular and intramyocyte spaces. Reduction in intramyocyte PO_2_ can be problematic because it promotes depletion of finite muscle phosphocreatine and glycogen stores, intracellular accumulation of metabolites (including ADP, Pi, and H^+^) and acid‐base perturbations associated with fatigue (Hogan et al., 1992; Wilson et al., 1977). Therefore, based on the above observations, potential impairments in contracting muscle capillary hemodynamics with GLI could result in pernicious consequences for oxidative metabolism and exercise tolerance (Colburn, Weber, et al., 2020; Lu et al., 2013).

Despite marked reductions in RBC hemodynamics (i.e., decreased *f*_RBC_ and *V*_RBC_; Figure 2), GLI did not change microvascular blood flow heterogeneity (as evaluated by the coefficient of variation of *f*_RBC_, *V*_RBC_, and Hct_cap_) in the resting spinotrapezius. Due to the nature of our experimental protocol (i.e., assessment of the same microcirculatory fields, and whenever possible, capillaries between conditions), these data indicate that any spatial vascular/metabolic mismatch present at rest was not exacerbated with GLI. Whether the same holds true for the contracting skeletal muscle (amid dynamic temporal and spatial changes in O_2_ delivery‐utilization) remains to be determined. Importantly, the consequences of microvascular blood flow heterogeneity assume greater significance during exercise when high O_2_ fluxes (which can rise >100‐fold compared to rest) are required to support oxidative metabolism.

### 4.3 Clinical implications

Sulfonylureas are the most popular second‐line treatment prescribed for type 2 diabetes mellitus (Montvida et al., 2018), promoting insulin release via inhibition of pancreatic K_ATP_ channels. However, systemic inhibition of K_ATP_ channels with these oral medications may also compromise key components of capillary gas exchange within skeletal muscle as revealed herein (i.e., lowered *f*_RBC_ and *V*_RBC_; Figure 2). The latter microvascular impairments compound with those described previously in the diabetic skeletal muscle (Kindig et al., 1998; Padilla et al., 2006) thus questioning the therapeutic value of sulfonylurea medications for diabetic patients. Systemic sulfonylurea administration has also been reported to reduce contracting muscle bulk blood flow (Holdsworth et al., 2015; Keller et al., 2004; Thomas et al., 1997) and submaximal and maximal exercise tolerance (Colburn, Weber, et al., 2020; Lu et al., 2013). Although no decrements in intracapillary DO_2_m with GLI were found herein at rest, potential reductions in the proportion of capillaries supporting continuous RBC flow with sulfonylurea medications during contractions would be expected to impair muscle glucose uptake (via reductions in exchange surface area). As such, exercise intolerance may be exacerbated in diabetes by systemic sulfonylurea medications impairing K_ATP_ channel regulation of skeletal muscle capillary hemodynamics.

### 4.4 Experimental considerations

Superfusion of the spinotrapezius muscle with GLI was employed herein to locally inhibit K_ATP_ channels *in vivo*. This topical drug delivery method was chosen to prevent the potential confound of alterations in central hemodynamics (e.g., MAP, HR, systemic vascular resistance), myocardial hemodynamics and function (e.g., coronary vascular conductance and O_2_ delivery, left ventricular relaxation rate), sympathetic nerve activity, visceral organ blood flow, blood insulin and glucose concentration, among others with systemic drug administration as reported previously (Colburn, Holdsworth, et al., 2020; Colburn, Weber, et al., 2020; Duncker et al., 2001; Farouque & Meredith, 2003a; Holdsworth et al., 2015, 2016; Rocha et al., 2020; Thomas et al., 1997). Nonetheless, it is not possible to pinpoint the site of GLI action (e.g., across the vascular network and/or among distinct cell types) with the current experimental protocol. Although our GLI concentration and superfusion protocol were based on previous microcirculatory studies (Cohen & Sarelius, 2002; Hammer et al., 2001; Hodnett et al., 2008; Jackson, 1993; Lu et al., 2013; Murrant & Sarelius, 2002; Saito et al., 1996; Xiang & Hester, 2009) as described earlier, this approach could lead to non‐specific vasodilation (Jiang et al., 2007; Tykocki et al., 2017) and potentially underestimate the contribution of K_ATP_ channels to microvascular control. It is noteworthy that, despite the latter considerations and the modest sample size (*n* = 6), significant GLI‐induced reductions in *f*_RBC_ and *V*_RBC_ were observed in the resting spinotrapezius muscle (Figures 2 and 3). Post hoc power calculations indicate that a total of 48 animals would be needed to detect significant changes in the %RBC‐flowing capillaries between conditions.

Previous evidence indicates that muscle capillaries increase in diameter by ~0.5–1.0 µm from their arteriolar to venular ends (Smaje et al., 1970). Although *d*_cap_ measurements herein were not normalized for the distance from arteriolar or venular ends, our *d*_cap_ assessment at random sites along the capillary length is not anticipated to introduce systematic errors. Moreover, capillary structural and hemodynamics analyses of the same microcirculatory fields within a given animal (and, in 47% of the cases, within the same capillary between CON and GLI conditions) likely minimized the potential for biases (including spatial vascular/metabolic heterogeneity, and heterogeneous *f*_RBC_, *V*_RBC_ and Hct_cap_ distributions) to be expressed in the present results. The lack of changes in *d*_cap_ in the face of reduced *f*_RBC_ and *V*_RBC_ with GLI (Figure 2) is consistent with upstream arteriolar regulation of skeletal muscle blood flow as mentioned above (Joyner & Casey, 2015; Kindig & Poole, 2001; Laughlin et al., 2012; Segal, 2005).

### 4.5 Summary and conclusions

Local inhibition of K_ATP_ channels with GLI did not reduce the proportion of capillaries supporting continuous RBC flow or Hct_cap_ in the resting rat spinotrapezius muscle. These data are indicative of preserved muscle O_2_ diffusing capacity (DO_2_m) with GLI. In contrast, GLI lowered both *f*_RBC_ and *V*_RBC_ thus impairing perfusive microvascular O_2_ transport (Q̇m) and lengthening RBC capillary transit times, respectively. Considering the interdependence between diffusive and perfusive O_2_ conductances (i.e., %O_2_ extraction determined largely by the local DO_2_m/Q̇m; vide Eq. 2 above) (Roca et al., 1992), such GLI‐induced alterations in capillary hemodynamics are expected to elevate muscle fractional O_2_ extraction to sustain a given metabolic rate. Taken together, the current results suggest that K_ATP_ channels regulate capillary hemodynamics (and, therefore, microvascular gas exchange) in resting skeletal muscle.

## DISCLOSURE

No conflicts of interest, financial or otherwise, are declared by the authors.

## AUTHOR CONTRIBUTIONS

DMH, TIM, and DCP conceived and designed research; DMH, AT, JCC, and TDC performed experiments; DMH analyzed data; DMH, TIM, and DCP interpreted results of experiments; DMH prepared figures and drafted manuscript; DMH, AT, JCC, TDC, TIM, and DCP edited and revised manuscript; DMH, AT, JCC, TDC, TIM, and DCP approved final version of the manuscript.
